# Assessing systemic and non-systemic transmission risk of tick-borne encephalitis virus in Hungary

**DOI:** 10.1371/journal.pone.0217206

**Published:** 2019-06-04

**Authors:** Kyeongah Nah, Felicia Maria G. Magpantay, Ákos Bede-Fazekas, Gergely Röst, Attila János Trájer, Xiaotian Wu, Xue Zhang, Jianhong Wu

**Affiliations:** 1 Department of Mathematics and Statistics, York University, Toronto, Ontario, Canada; 2 Department of Mathematics and Statistics, Queen’s University, Kingston, Ontario, Canada; 3 Institute of Ecology and Botany, MTA Centre for Ecological Research, Vácrátót, Hungary; 4 GINOP Sustainable Ecosystems Group, MTA Centre for Ecological Research, Tihany, Hungary; 5 Wolfson Centre for Mathematical Biology, University of Oxford, Oxford, United Kingdom; 6 Bolyai Institute, University of Szeged, Szeged, Hungary; 7 Department of Limnology, University of Pannonia, Veszprém, Hungary; 8 Institute of Environmental Engineering, University of Pannonia, Veszprém, Hungary; 9 College of Arts and Sciences, Shanghai Maritime University, Shanghai, China; 10 Department of Mathematics, Northeastern University, Shenyang, China; Tufts University Cummings School of Veterinary Medicine, UNITED STATES

## Abstract

Estimating the tick-borne encephalitis (TBE) infection risk under substantial uncertainties of the vector abundance, environmental condition and human-tick interaction is important for evidence-informed public health intervention strategies. Estimating this risk is computationally challenging since the data we observe, i.e., the human incidence of TBE, is only the final outcome of the tick-host transmission and tick-human contact processes. The challenge also increases since the complex TBE virus (TBEV) transmission cycle involves the non-systemic route of transmission between co-feeding ticks. Here, we describe the hidden Markov transition process, using a novel TBEV transmission-human case reporting cascade model that couples the susceptible-infected compartmental model describing the TBEV transmission dynamics among ticks, animal hosts and humans, with the stochastic observation process of human TBE reporting given infection. By fitting human incidence data in Hungary to the transmission model, we estimate key parameters relevant to the tick-host interaction and tick-human transmission. We then use the parametrized cascade model to assess the transmission potential of TBEV in the enzootic cycle with respect to the climate change, and to evaluate the contribution of non-systemic transmission. We show that the TBEV transmission potential in the enzootic cycle has been increasing along with the increased temperature though the TBE human incidence has dropped since 1990s, emphasizing the importance of persistent public health interventions. By demonstrating that non-systemic transmission pathway is a significant factor in the transmission of TBEV in Hungary, we conclude that the risk of TBE infection will be highly underestimated if the non-systemic transmission route is neglected in the risk assessment.

## Introduction

Tick-borne encephalitis (TBE), an arboviral infection of the central nervous system caused by bites from infected ticks, has been a major public health concern for much of Central Europe. In certain parts of Central Europe (e.g. in the Czech Republic) the occurrence of TBE is increasing since climate change pushes the vector to the higher altitudes [1, 2]. The risk of human infection depends on the prevalence of the virus spread in the tick-host ecological cycle, and on the tick-human contact that is effective for the disease transmission. Evaluating the human infection needs accurate estimation of the tick population dynamics which is highly regulated by the environmental and climatic conditions [3–8]; the effectiveness of pathogen transmission per tick-host contact, and the human case reporting given infection probability which is quite stochastic [9]. All of these involve substantial uncertainties in ecological, epidemiological and environmental parameters.

In the affected countries of the European Union, TBE incidence data is available and indicates the disease trend. For example, in Hungary TBE cases have been reported to National Database of Epidemiological Surveillance System since 1977. Between 1977 to 1996, the average annual incidence was approximately 2.7 per 100,000, and this incidence has shown dramatic decrease since 1997. Multiple factors may have contributed to this decrease, including under-reporting followed by decreased serological examination, or public vaccination in early 1990s [10, 11]. Whether or not the decrease in the incidence reflects the actual decrease in human TBE infection level, it seems probable that TBE infection level has not been decreased in the ecological cycle. The studies on the seropositivity of animals in 1960 to 1970 and 2005 support this argument [12, 13].

One of our goals is to develop a mathematical model to infer the TBEV transmission level in the ecological cycle. In particular, we aim to use a mathematical model fitting the surveillance data to determine whether there was significant increase in the basic reproduction number of TBE, and whether there was significant decrease in the expected TBE cases between 1980 to 2015.

There may be substantial amount of unreported cases, since many TBE infections go undiagnosed or unreported [10, 14]. Therefore, estimation of the TBE infection risk under significant uncertainties of the tick-host interaction and tick-human transmission process is challenging since the data we observe, i.e., human incidence of TBE, is only the final outcome of the tick-host transmission, tick-human contact and the case reporting upon infection. To meet this challenge, here we develop a coupled TBEV transmission-human report cascade model, that consists of two important components: the TBEV transmission dynamics among ticks, animal hosts and humans, and the stochastic process of TBE reporting given human TBE infection. We will use this coupled cascade model to facilitate fitting the human TBE incidence data to the model in generating good estimation of key parameters including seasonal human-tick encounter rates and human case reporting probability.

TBEV transmission involve two important routes, systemic and non-systemic transmission. Transovarial transmission is possible, but it only occurs at a low frequency [15]. Systemic transmission of TBEV involves infected ticks and vertebrate hosts. The tick *Ixodes ricinus* is the vector of the European TBEV subtype [16]. The *Ixodes* ticks undergo complex developmental cycle involving egg, larva, nymph and adult stages and the full life cycle takes average of 3 years [17]. Ticks take blood meals from hosts to develop from one stage to the next stage. At each blood meal, ticks are integrated into the epidemiological chain of the virus transmission. Systemic transmission of TBEV has the following cycle: hosts acquire infection from infected nymphs. The infected hosts pass the virus to feeding larvae. As the infected larvae develop into nymphs, the nymphs can again transmit virus into a new host. Several mathematical models, see for example [16, 18–21], have been developed and analyzed to examine the ecological or epidemiological factors that govern the abundance of *Ixodes ricinus* ticks or TBE infections. However, only few studies have focused on the significance of the non-systemic route of TBEV transmission [22–24], through which a susceptible vector can acquire the infection by co-feeding with infected vectors on the same host [25–29] even when the pathogen has not established within the host for systemic transmission. It was observed that TBE in Europe may be mainly maintained by non-systemic transmission between co-feeding ticks [30], and an early modeling study [23] shows that the basic reproduction number (*R*_0_) of TBE without non-systemic route of transmission is estimated to be less than 1, which means that TBE would not persist without non-systemic route of transmission. In comparison, the work of [31] shows that systemic transmission cycle alone can sustain the transmission of TBEV in a natural focus. Both systemic and non-systemic transmissions, as well as the vector abundance, are heavily influenced by the climatic and environmental conditions, which are characterized by uncertainty and seasonal variations. Some recently developed stage-structured tick population and tick-borne diseases (Lyme disease in particular) models [20, 32, 33] have used these environmental condition data. However, because of uncertainties in some of the parameters in the aforementioned models, and in the consideration of both systemic and non-systematic transmission with the co-feeding transmission efficacy unsettled, we will need not only refine existing model structure but also tune and estimate some of the model parameters by fitting a disease transmission model to time series incidence data [34, 35].

Data fitting is an important issue in TBE modeling, since human is only a dead-end host of TBEV transmission [36], and can be infected by bites of both infected nymphs and female ticks, although rarely, consumption of unpasteurized milk from infected animals can also cause TBE infection [37]. In addition, two-thirds of human TBEV infections are either sub-clinical or asymptomatic [38], suggesting that there may be more infected cases than cases reported, therefore the probability of reporting given infection needs to be estimated in order to accurately evaluate the TBE risk in the population.

In our developed TBEV transmission-human report cascade model, we couple the TBEV (systemic and non-systemic) transmission dynamics among ticks and animal hosts with the stochastic process of TBE reporting given human TBE infection. We will use this coupled system and the TBE incidence data, together with climate data to estimate such key epidemiological parameters as seasonal human-tick encounter rate, case reporting probability, tick-host contact for disease transmission, and tick-human transmission. With these key parameters and probabilities estimated, we can use our coupled system to estimate the basic reproduction numbers of TBE in Hungary, evaluate the transmission potential of TBEV in the enzootic cycle along with the climate change, and assess the significance of non-systemic pathway in the transmission of TBEV in Hungary.

## Materials and methods

We introduce a tick-host and tick-human transmission cascade model with two integrated parts to describe the dynamical relationship between the epidemic in the tick-host population and the subsequent tick-to-human infections. It is a hidden Markov Model with a transition process characterized by a deterministic TBEV (tick-host) transmission model among tick and host populations and an observation process described by a stochastic model of human TBE reporting given infection.

### TBEV transmission model among ticks and hosts

Our TBEV transmission model describes the seasonal transmission of a pathogen among ticks and hosts with a system of ordinary differential equations with periodic coefficients, modified from some earlier work of modeling Lyme disease dynamics [32, 33] but adapted for some unique features of TBEV dynamics including in particular co-feeding transmission. We will first stratify the tick population and host population into susceptibles and infecteds, depending on the state of infection; we further stratify the tick population by their physiological stages.

#### Stages and development

Ticks in questing stages move to engorged stages upon successful host-feeding. Nymphal and larval *Ixodes ricinus* feed on small-to-medium-sized mammals such as rodents, while adult ticks feed on large-sized mammals [39, 40]. Engorged adults are reproducible and lay eggs while engorged larvae and nymphs move into the next stages after completing the maturation process. As these reproduction and development processes involve a few key biological activities which are highly relevant to the host density and environment conditions, we need to further stratify the tick population within each physiological stage. Namely, we need to consider the following activity states of tick population: questing larvae (*L*_*q*_), engorged larvae (*L*_*e*_), questing nymphs (*N*_*q*_), engorged nymphs (*N*_*e*_), questing adults (*A*_*q*_), engorged adults (*A*_*e*_) and eggs (*E*).

Eggs develop into the questing larval stage with the developmental rate *d*_*el*_(*t*). Questing larvae attach to hosts with rate *α*_*l*_(*t*). Among them, only the proportion (*f*_*l*_) who survive the feeding stage move to the engorged larval stage. Once the engorged larvae completes maturation they are accounted as questing nymphs. The same process is repeated from questing nymphs to engorged nymphs and from questing adults to engorged adults. Then, the female engorged adults lay eggs with oviposition rate *d*_*pop*_(*t*). A parameter *η* refers to the proportion of engorged female adults to engorged adults. We use Ricker function to describe the birth rate of eggs, with the parameter *p* being the maximal egg-laying rate and the parameter *ω* representing the degree of density dependence in fecundity [41]. Each stages have distinct mortalities (*μ*_*e*_, *μ*_*ql*_, *μ*_*el*_, *μ*_*qn*_, *μ*_*en*_, *μ*_*qa*_, *μ*_*ea*_).

#### Seasonal dependence

Developmental rates and the host-attaching rates have strong seasonal dependence and those parameters are thus time-dependent and functions of the independent time variable *t*. Other parameters showing less seasonal dependence will be assumed to be constant. Since the tick questing activity depends on a climatic condition [42], we decompose the host-attaching rates (*α*_*l*_(*t*), *α*_*n*_(*t*) and *α*_*a*_(*t*)) into the proportion of actively searching ticks among questing ticks at time *t* (*p*_*l*_(*t*), *p*_*n*_(*t*) and *p*_*a*_(*t*)) (and here and in what follows, subindices *l*, *n* and *a* are indicating these parameters are relevant to the particular stages of larvae, nymphs and adults respectively) and the host-finding rate of those actively searching ticks (λ_*l*_, λ_*n*_ and λ_*a*_). That is,

$$\begin{array}{c} \begin{array}{cc} \alpha_{l}\left(t\right) & =p_{l}\left(t\right)\times \lambda_{l}\\ \alpha_{n}\left(t\right) & =p_{n}\left(t\right)\times \lambda_{n}\\ \alpha_{a}\left(t\right) & =p_{a}\left(t\right)\times \lambda_{a}. \end{array} \end{array}$$

#### Tick population growth (ecological) model

We can now formulate a system of ordinary differential equations for the tick population growth (ecological) model:

$$\begin{array}{c} \left\{ \begin{array}{cc} L_{q}^{\prime }\left(t\right) & =d_{el}\left(t\right)E\left(t\right)-\alpha_{l}\left(t\right)L_{q}\left(t\right)-\mu_{ql}L_{q}\left(t\right),\\ L_{e}^{\prime }\left(t\right) & =f_{l}\alpha_{l}\left(t\right)L_{q}\left(t\right)-d_{ln}\left(t\right)L_{e}\left(t\right)-\mu_{el}L_{e}\left(t\right),\\ N_{q}^{\prime }\left(t\right) & =d_{ln}\left(t\right)L_{e}\left(t\right)-\alpha_{n}\left(t\right)N_{q}\left(t\right)-\mu_{qn}N_{q}\left(t\right),\\ N_{e}^{\prime }\left(t\right) & =f_{n}\alpha_{n}\left(t\right)N_{q}\left(t\right)-d_{na}\left(t\right)N_{e}\left(t\right)-\mu_{en}N_{e}\left(t\right),\\ A_{q}^{\prime }\left(t\right) & =d_{na}\left(t\right)N_{e}\left(t\right)-\alpha_{a}\left(t\right)A_{qs}\left(t\right)-\mu_{qa}A_{qs}\left(t\right),\\ A_{e}^{\prime }\left(t\right) & =f_{a}\alpha_{a}\left(t\right)A_{q}\left(t\right)-d_{pop}\left(t\right)A_{e}\left(t\right)-\mu_{ea}A_{e}\left(t\right),\\ E^{\prime }\left(t\right) & =p\cdot \eta d_{pop}\left(t\right)A_{e}\left(t\right)\cdot e^{-\omega \cdot \eta d_{pop}\left(t\right)A_{e}\left(t\right)}-d_{el}\left(t\right)E\left(t\right)-\mu_{e}E\left(t\right). \end{array}\right. \end{array}$$ (1)

while the list of variables appear in Table 1, and the full list of the parameters appears in Table 2. The number of egg-laying adults is implicitly accounted in the model, see S1 Appendix for the details.

**Table 1:** Variables.

Variable	Definition
*E*	Number of eggs
*L*_*q*_	Number of questing larvae
*L*_*es*_ [*L*_*ei*_]	Number of susceptible [infected] engorged larvae
*N*_*qs*_ [*N*_*qi*_]	Number of susceptible [infected] questing nymphs
*N*_*es*_ [*N*_*ei*_]	Number of susceptible [infected] engorged nymphs
*A*_*qs*_ [*A*_*qi*_]	Number of susceptible [infected] questing adults
*A*_*e*_	Number of engorged adults
*H*_*s*_ [*H*_*i*_]	Number of of susceptible [infected] rodents

**Table 2:** List of parameters.

Parameters	Description
*d*_*el*_, *d*_*ln*_, *d*_*na*_	development rate from eggs to larvae, engorged larvae to nymphs, engorged nymphs to adults
*d*_*pop*_	development rate from engorged adults to egg-laying adults
*f*_*l*_, *f*_*n*_, *f*_*a*_	probability of successful feeding for host-attached larvae, nymphs and adults
*α*_*l*_, *α*_*n*_, *α*_*a*_	host-attaching rates for questing larvae, nymphs and adults
*p_l_*, *p_n_*, *p_a_*	proportion of active ticks among questing larvae, nymphs and adults
λ_*l*_, λ_*n*_, λ_*a*_	host-attaching rate of active questing larvae, nymphs and adults
*p*	maximum number of eggs laid by egg-laying adult female ticks
*η*	proportion of engorged female adults to engorged adults
*ω*	degree of density dependence in fecundity
*μ*_*ql*_, *μ*_*qn*_, *μ*_*qa*_	mortality rate of questing larvae, nymphs and adults
*μ*_*el*_, *μ*_*en*_, *μ*_*ea*_	mortality rate of engorged larvae, nymphs and adults
*μ*_*e*_	mortality rate of eggs
*b*	mortality rate of hosts
*γ*	recovery rate of infected hosts
*β*_*hl*_, *β*_*hn*_, *β*_*nh*_	transmission efficacy from hosts to larvae, hosts to nymphs, nymphs to hosts
*T*_*f*_	average duration of feeding
*c*	probability of an infected nymph to induce non-systemic infection to the cofeeding susceptible larvae
*m_l_*	minimum temperature for the coincidence of host availability and the activity of questing larvae

#### Transmission diagram and systemic transmission

**Fig 1:**
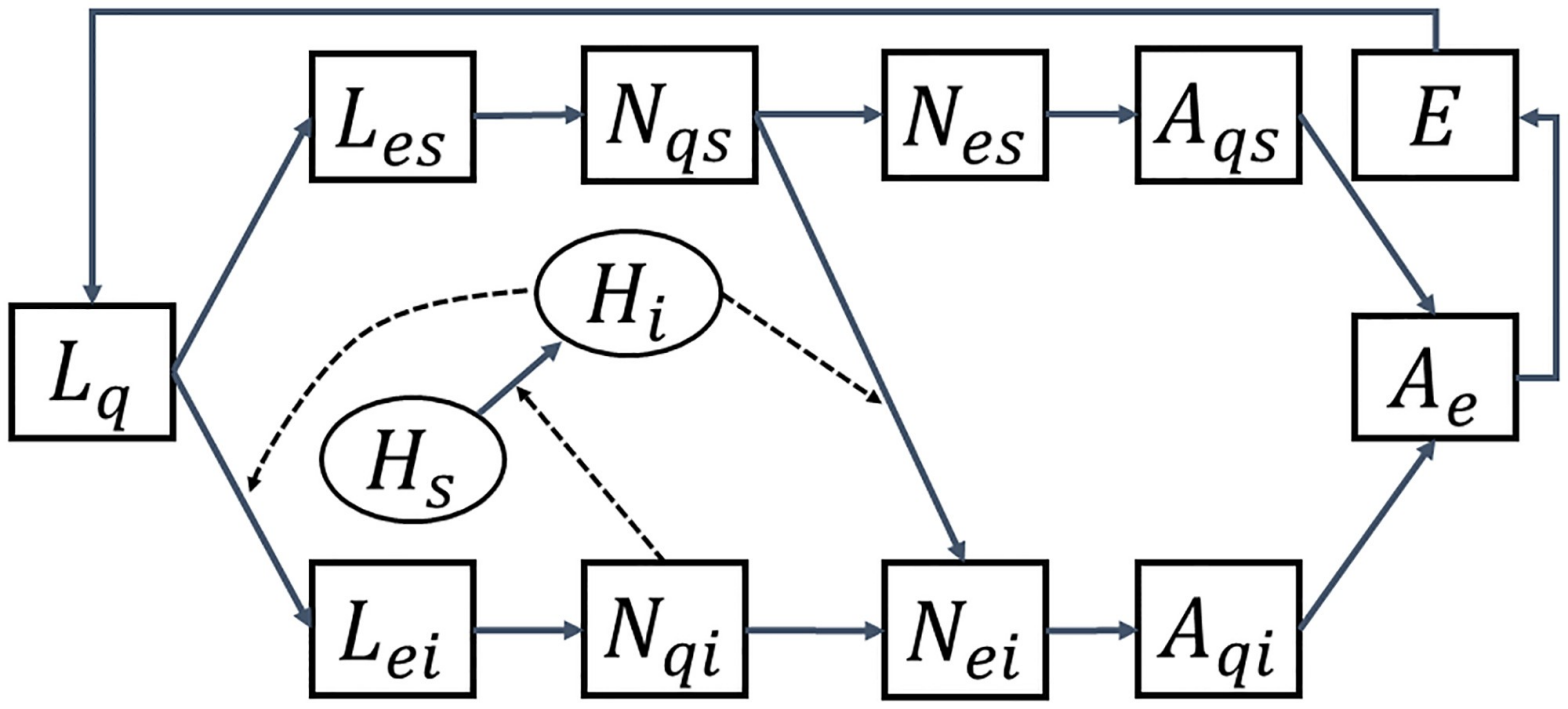
An illustration of the systemic TBEV transmission involving ticks in different physiological stages and a competent host. Dashed lines depict infection routes. Tick population is stratified by the stages: questing larvae (*L*_*q*_), engorged larvae (*L*_*e*_), questing nymphs (*N*_*q*_), engorged nymphs (*N*_*e*_), questing adults (*A*_*q*_), engorged adults (*A*_*e*_) and eggs (*E*). The additional subscripts *s* and *i* refer to susceptible and infecteds, respectively. Host population is divided into susceptible (*H*_*s*_) and infecteds (*H*_*i*_). The variables are described in Table 1.

Fig 1 illustrates some major epidemiological processes that must be incorporated into our transmission dynamics model. Transovarial infection (transmission from infected females to their eggs) for TBEV occurs only rarely, so we will not consider this vertical transmission route, and hence there is no infection by questing larvae stage [43]. Since we ignore the vertical transmission, there is no need to divide the engorged adult population into susceptible and infecteds. In the systemic transmission route, questing ticks acquire viruses by feeding infected hosts, and infected ticks can also pass the virus to susceptible hosts during feeding. Note that the hosts for adult ticks can also be infected and pass the virus via untreated dairy products, however, TBE is mainly transmitted by tick bite and thus not incorporated explicitly in our model system [44]. Questing adults are further stratified into susceptibles and infecteds since questing adults involve in the human infection. In what follows, the standard incidence is used to describe the systemic transmission of TBEV through the contact of a susceptible host (tick) with an infected tick (host).

#### Co-feeding transmission

Our main focus is on how to model the transmission of TBEV between co-feeding ticks [26, 45]. Let *δ*(*N*_*qi*_(*t*), *H*) be the probability of a susceptible feeding tick being infected by co-feeding nymphs during which the ticks are co-feeding a host. Let *c* be the probability of an infected nymph to induce non-systemic infection to the co-feeding susceptible tick. We assume that the events of each infected nymph triggering non-systemic infection to co-feeding larvae are independent. Then, the probability of a larvae to get non-systemic infection while it is co-feeding with *n* number of infected nymphs is

$$\begin{array}{c} 1-{\left(1-c\right)}^{n}. \end{array}$$ (2)

The number of feeding infected nymphs at time *t* is

$$\begin{array}{c} \int_{t-T_{f}}^{t}\alpha_{n}\left(u\right)N_{qi}\left(u\right)e^{-\mu_{{}_{fn}}\left(t-u\right)}du, \end{array}$$

where *T*_*f*_ is the average duration of feeding and $\mu_{{}_{fn}}$ is the mortality of feeding infected nymphs. Considering that the duration of feeding is relatively short, we estimate the average number of feeding infected nymphs per host at time *t* with

$$\begin{array}{c} T_{f}\alpha_{n}\left(t\right)N_{qi}/H. \end{array}$$

From the above assumptions, we have the following non-linear co-feeding probability formulation:

$$\begin{array}{c} \delta \left(N_{qi}\left(t\right),H\right)=1-{\left(1-c\right)}^{T_{f}\alpha_{n}\left(t\right)N_{qi}/H}. \end{array}$$

Non-systemic transmission can also happen when the host is immune to the infection [46]. Therefore, the force of infection for the questing larvae through non-systemic transmission route is

$$\begin{array}{c} \delta \left(N_{qi}\left(t\right),H\right)(\left(1-\beta_{hl}\right)\frac{H_{i}\left(t\right)}{H}+\frac{H-H_{i}\left(t\right)}{H})f_{l}\alpha_{l}\left(t\right). \end{array}$$

This formulation captures the fact that non-systemic transmission is only possible when both infective ticks and susceptible ticks are actively questing [7].

By the above discussions, the tick population growth model (1) can be generalized to the following TBEV transmission dynamics among the relevant hosts and ticks:

$$\begin{array}{c} \left\{ \begin{array}{cc} L_{q}^{\prime }\left(t\right) & =d_{el}\left(t\right)E\left(t\right)-\alpha_{l}\left(t\right)L_{q}\left(t\right)-\mu_{ql}L_{q}\left(t\right),\\ L_{es}^{\prime }\left(t\right) & =\left(1-\delta \left(N_{qi}\left(t\right),H\right)\right)(\left(1-\beta_{hl}\right)\frac{H_{i}\left(t\right)}{H}+\frac{H-H_{i}\left(t\right)}{H})f_{l}\alpha_{l}\left(t\right)L_{q}\left(t\right)\\ & -d_{ln}\left(t\right)L_{es}\left(t\right)-\mu_{el}L_{es}\left(t\right),\\ L_{ei}^{\prime }\left(t\right) & =\delta \left(N_{qi}\left(t\right),H\right)(\left(1-\beta_{hl}\right)\frac{H_{i}\left(t\right)}{H}+\frac{H-H_{i}\left(t\right)}{H})f_{l}\alpha_{l}\left(t\right)L_{q}\left(t\right)\\ & +\beta_{hl}f_{l}\alpha_{l}\left(t\right)L_{q}\left(t\right)\frac{H_{i}\left(t\right)}{H}-d_{ln}\left(t\right)L_{ei}\left(t\right)-\mu_{el}L_{ei}\left(t\right),\\ N_{qs}^{\prime }\left(t\right) & =d_{ln}\left(t\right)L_{es}\left(t\right)-\alpha_{n}\left(t\right)N_{qs}\left(t\right)-\mu_{qn}N_{qs}\left(t\right),\\ N_{qi}^{\prime }\left(t\right) & =d_{ln}\left(t\right)L_{ei}\left(t\right)-\alpha_{n}\left(t\right)N_{qi}\left(t\right)-\mu_{qn}N_{qi}\left(t\right),\\ N_{es}^{\prime }\left(t\right) & =\left(1-\delta \left(N_{qi}\left(t\right),H\right)\right)(\left(1-\beta_{hn}\right)\frac{H_{i}\left(t\right)}{H}+\frac{H-H_{i}\left(t\right)}{H})f_{n}\alpha_{n}\left(t\right)N_{qs}\left(t\right)\\ & -d_{na}\left(t\right)N_{es}\left(t\right)-\mu_{en}N_{es}\left(t\right),\\ N_{ei}^{\prime }\left(t\right) & =\delta \left(N_{qi}\left(t\right),H\right)(\left(1-\beta_{hn}\right)\frac{H_{i}\left(t\right)}{H}+\frac{H-H_{i}\left(t\right)}{H})f_{n}\alpha_{n}\left(t\right)N_{qs}\left(t\right)\\ & +\beta_{hn}f_{n}\alpha_{n}\left(t\right)N_{qs}\left(t\right)\frac{H_{i}\left(t\right)}{H}+f_{n}\alpha_{n}\left(t\right)N_{qi}\left(t\right)-d_{na}\left(t\right)N_{ei}\left(t\right)-\mu_{en}N_{ei}\left(t\right),\\ A_{qs}^{\prime }\left(t\right) & =d_{na}\left(t\right)N_{es}\left(t\right)-\alpha_{a}\left(t\right)A_{qs}\left(t\right)-\mu_{qa}A_{qs}\left(t\right),\\ A_{qi}^{\prime }\left(t\right) & =d_{na}\left(t\right)N_{ei}\left(t\right)-\alpha_{a}\left(t\right)A_{qi}\left(t\right)-\mu_{qa}A_{qi}\left(t\right),\\ A_{e}^{\prime }\left(t\right) & =f_{a}\alpha_{a}\left(t\right)\left(A_{qs}\left(t\right)+A_{qi}\left(t\right)\right)-d_{pop}\left(t\right)A_{e}\left(t\right)-\mu_{ea}A_{e}\left(t\right),\\ E^{\prime }\left(t\right) & =p\cdot \eta d_{pop}\left(t\right)A_{e}\left(t\right)\cdot e^{-\omega \cdot \eta d_{pop}\left(t\right)A_{e}\left(t\right)}-d_{el}\left(t\right)E\left(t\right)-\mu_{e}E\left(t\right),\\ H_{s}^{\prime }\left(t\right) & =bH-\beta_{nh}\alpha_{n}\left(t\right)\frac{N_{qi}\left(t\right)}{H}H_{s}\left(t\right)-bH_{s}\left(t\right),\\ H_{i}^{\prime }\left(t\right) & =\beta_{nh}\alpha_{n}\left(t\right)\frac{N_{qi}\left(t\right)}{H}H_{s}\left(t\right)-\gamma H_{i}\left(t\right)-bH_{i}\left(t\right). \end{array}\right. \end{array}$$ (3)

The list of variables appear in Table 1, and the full list of the parameters appears in Table 2.

Because of the limited data on the host population, we remove explicit dependence on the parameter *H* from the model system by normalizing other variables with *H*. The normalized system is given in S1 Appendix.

#### TBE human infection and reporting model

Most *I. ricinus* tick bites to human are from ticks in nymphal and adult stages [47]. In our model, we assume that human can be infected with TBEV by the bites of infected nymphs and infected adult ticks. The number of newly infected cases between time *t* − Δ and *t* is given by

$$\begin{array}{c} \Delta I_{H}\left(t-\Delta ,t\right)=\int_{t-\Delta }^{t}(\Lambda_{n}\left(u\right)p_{n}\left(u\right)N_{qi}\left(u\right)+\Lambda_{a}\left(u\right)p_{a}\left(u\right)A_{qi}\left(u\right))du, \end{array}$$ (4)

where Λ_*n*_(*t*) and Λ_*a*_(*t*) are the temperature-dependent human-attaching rate of nymphs and adults,

$$\begin{array}{c} \Lambda_{n}\left(t\right)=\kappa \alpha e^{0.058\cdot T(t)},\\ \Lambda_{a}\left(t\right)=\alpha e^{0.058\cdot T(t)}, \end{array}$$

adapting the temperature related human outdoor activity in Hungary [48]. Here, the parameter *α* representing a degree of temperature dependency on the human-attaching rate of nymphs will be estimated from the surveillance data, and *κ* (also a parameter to be estimated) is the relative ratio between nymphs and adults for the attachments to the human.

We denote this number of newly infected cases between time *t* − Δ and *t* by *I*_*t*_, with Δ being the reporting period. We assume that *C*_*t*_, the number of cases reported during reported period Δ follows a negative binomial distribution with mean *ρI*_*t*_ and variance $I_{t}+\tau^{2}I_{t}^{2}$, where *ρ* is the reporting probability and *τ* stands for the overdispersion parameter. Again, these parameters will be estimated using the parameter identification procedures described below, when other parameters, listed in Tables 3 and 4 are used. The transmission efficacy of non-systemic transmission from a single infected nymph (*c*) is estimated from the equation 1 − (1 − *c*)^2^ = 0.65 in (2), where 0.65 is the probability of transmission of TBEV when co-feeding with two infected ticks [46]. Indeed, it is empirically observed that non-systemic transmission of TBEV in non-viraemic host is less efficient than transmission in viraemic host [25], as we see from our estimates, *c* < *β*_*hl*_. According to [6], the questing nymph activity is observed when the daily maximal temperature was over the 7°C. We assume that the questing nymph is inactive in a temperature below 7°C.

**Table 3:** Constant parameter values.

Notation	Value (unit)	Refs.	Notation	Value (unit)	Refs.
*p*	2000 (egg)	[39, 49, 50]	*η*	0.5	[51]
*μ*_*e*_	0.02618 (day^−1^)	[49]	*μ*_*ql*_	0.0068 (day^−1^)	[49]
*μ*_*el*_	0.001428 (day^−1^)	[49]	*μ*_*qn*_	0.0034 (day^−1^)	[49]
*μ*_*en*_	0.000476 (day^−1^)	[49]	*μ*_*qa*_	0.00136 (day^−1^)	[49]
*μ*_*ea*_	0.000408 (day^−1^)	[49]	*β*_*nh*_	0.9	[52]
*c*	0.4	[46]	*β*_*hl*_	0.8	[52]
*β*_*hn*_	0.8	[52]	*T*_*f*_	4 (day)	[53]

**Table 4:** Temperature dependent parameter values.

Parameters	Dependence on temperature *T* (°C) (unit)	Ref.
*d*_*el*_(*T*)	−0.0000112*T*^2^ + 0.002305*T* − 0.0185 (*T* ≥ 8.4) (day^−1^)	[3, 8]
*d*_*ln*_(*T*)	0.0000303*T*^2^ + 0.000733*T* − 0.00706 (*T* ≥ 7.4) (day^−1^)	[3, 8]
*d*_*na*_(*T*)	−0.00000796*T*^2^ + 0.00193*T* − 0.0161 (*T* ≥ 8.7) (day^−1^)	[3, 8]
*d*_*pop*_(*T*)	−0.00001867*T*^3^ + 0.0008724*T*^2^ − 0.006195*T* + 0.01802	[3]
	(*T* ≥ 4) (day^−1^)	
*p*_*l*_(*T*)	$\{\begin{array}{ll} 0 & T<m_{l}\\ 1 & T\geq m_{l} \end{array}$	[6]
*p*_*n*_(*T*), *p*_*a*_(*T*)	$\{\begin{array}{ll} 0 & T<7\\ 1 & T\geq 7 \end{array}$	[6]

## Data fitting and results

We use weekly mean temperature data between 1901 and 2015 obtained from a weather station in Szombathely (coordinates: 47.20N, 16.65E, 201.0m) in Vas County [54]. Vas region is one of the highly endemic area in Hungary [55]. Weekly human TBE incidence data from 1998 to 2008 is obtained from National Epidemiological Center of Hungary. The clinically diagnosed cases confirmed by laboratory serological test are counted as the reported TBE case.

With given parameter values in Tables 3 and 4 and temperature data, we fit human reporting model (4) with TBE incidence data between 1998 and 2008. The climate data between 1901-1998 was used for the initialization of the model, as explained in detail in S1 Appendix. By the maximum likelihood estimation, we estimate the unknown parameters: probabilities of successful feeding (*f*_*l*_, *f*_*n*_, *f*_*a*_), host-attaching rate of actively questing ticks (λ_*l*_, λ_*n*_, λ_*a*_), degree of density dependent fecundity (*ω*), host recovery rate (*γ*), mortality of hosts (*b*), relative ratio between nymphs and adults for the human attachments (*κ*), the degree of temperature dependency on the human-attaching rate of nymphs (*α*), the minimum temperature for the activity of questing larvae (*m_l_*) and the reporting probability (*ρ*). The maximum likelihood estimation was performed by the R package pomp, using trajectory matching [56, 57]. Using Latin hypercube sampling, we choose 10^4^ number of initial set of parameters and compared the likelihoods at each parameters set. We repeat the sampling process at the parameter sets with the maximum likelihoods. The convergence is checked by computing the likelihood profiles (S1 Appendix) over each of the unknown parameters.

**Fig 2:**
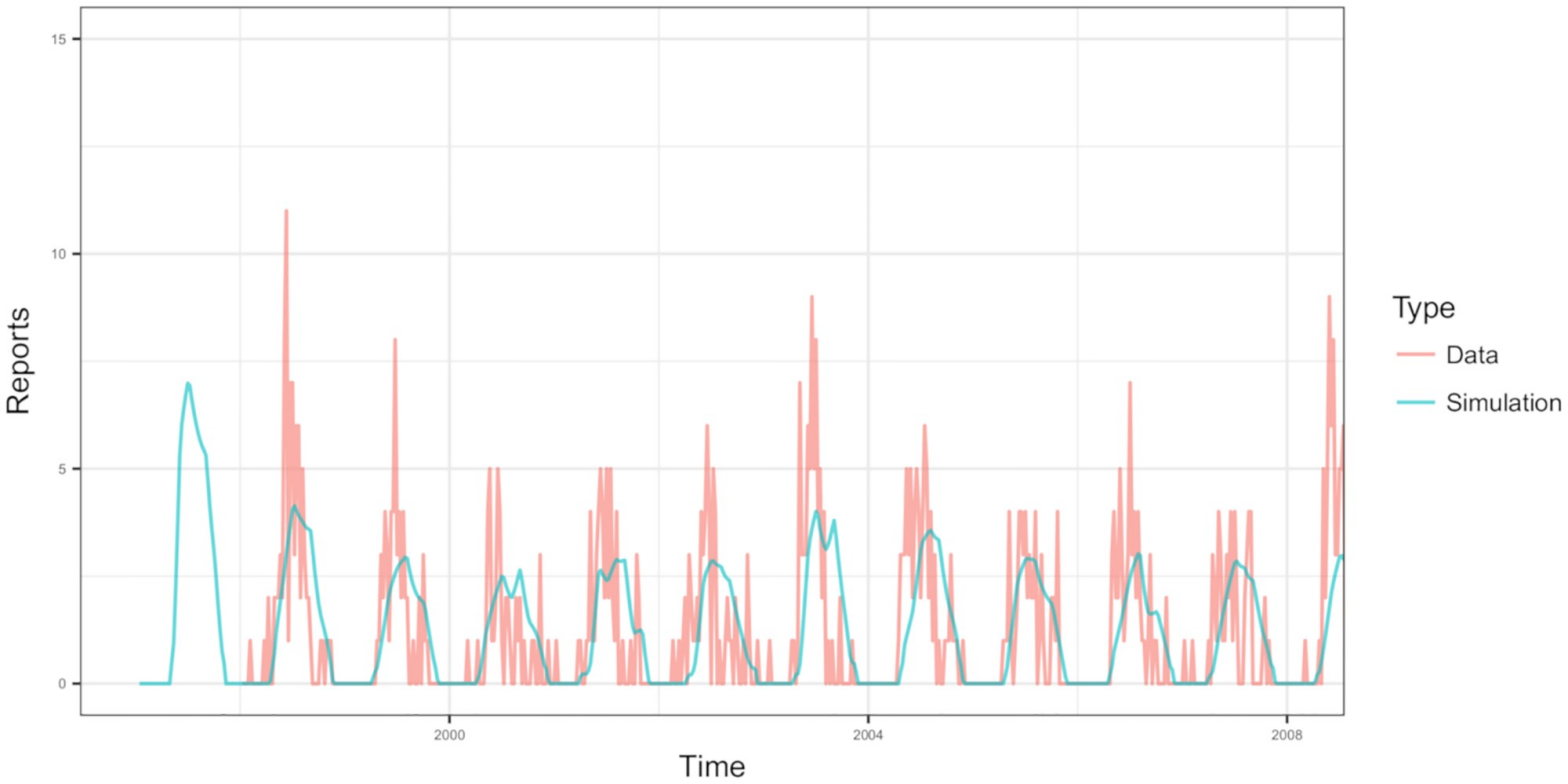
1998-2008 TBE case reports in Hungary and a model fit. Red line shows the monthly accumulation of the reported case data and skyblue line shows the sample simulation of the case report model at the maximal likelihood estimation. Fixed parameter values and estimated parameter values used for simulation are given in Tables 3 and 5.

Fig 2 shows a model fit and the 1998–2008 TBE case reports in Hungary. The sample simulation of the case report model at the maximal likelihood estimation depicts the binomial curve, which is known to be the characteristic of the TBE incidence in Hungary as well as many other European countries [58]. With the set of parameter values listed in Table 5, we obtain the parameterized model for TBEV transmission. All these values are in the 95% interval that yields the highest likelihood values. The model is then validated by the comparison of the model estimators with the data obtained in the observational studies.

**Table 5:** Parameter values used in the simulation.

Notation	Value (unit)	Notation	Value (unit)
*ω*	6.2 (per egg-laying adults)	*b*	0.0027 (day^−1^)
*γ*	0.1 (day^−1^)	*α*	1660 (day^−1^)
*f*_*l*_	0.19	*f*_*n*_	0.96
*f*_*a*_	0.81	λ_*l*_	0.0064 (day^−1^)
λ_*n*_	0.0014 (day^−1^)	λ_*a*_	24 (day^−1^)
*m_l_*	17 (°C)	*κ*	0.00004
*ρ*	0.64	*τ*	0.00003

### Prevalence of TBE in small mammals

According to the recent field study of [59, 60], 3.7% to 20.5% of rodents collected in Hungary during 2010-2013 were seropositive. In comparison, the annual average of infected rodents calculated from our parameterized model is 18.6% of the total rodents. For this estimation, we used the annual cycles of temperature data in Szombathely from 2010 to 2013 [54].

### Reporting probability of human TBE infecteds

More than two-thirds of TBE cases are known to be asymptomatic [61]. In the observation of [62], 30–50% of the studied tick-borne disease cases remembered a tick bite prior to the disease onset. For these reasons, the reported case of TBE is expected to be lower than the actual number of infected case. In comparison, the reporting probability (*ρ*) through our data fitting is estimated to be 0.64.

### Stage-dependent frequency of attachment to human

Estimated ratio of human-attaching rate for nymphs to the human-attaching rate for adults (*κ*) is less than 1, indicating shorter human questing time for adult ticks to nymphs.

### Duration of host infectivity

Estimated recovery rate of hosts is 0.1 leading to the average duration of the host infectivity as 10 days, while the experimentally-observed duration of host infectivity to ticks is known to be 2 to 3 days [26].

### Probability of surviving the feeding stage

From our model, the probability of successful feeding for host-attached larvae (*f*_*l*_) and nymphs (*f*_*n*_) are estimated to be 0.19 and 0.96, respectively. According to the experimental study on the infestation of *Ixodes ricinus*, 90 to 99 percent of nymphs and 46 to 72 percent of larvae has successfully fed the mice in the laboratory setting [53].

### Temperature dependence of the questing activity for larvae

In our model fitting, minimum temperature for the coincidence of host availability and the activity of questing larvae (*m*_*l*_) is estimated to be 17°C. This coincides with the average temperature of Szombathely in May, when the larvae starts to be detected in the field [63]. As *m*_*l*_ also depends on the host abundance, this estimated is larger than the minimum temperature for the larval ticks to become active. We also note that larvae of *Ixodes ricinus* are observed to have the normal activity between 15°C-27°C [64].

### Basic reproduction number

The basic reproduction number (*R*_0_) is a universally-recognized metric of the capacity of a parasite or a pathogen to reproduce given particular environmental conditions. We can use our parametrized model to evaluate the transmission potential of TBEV in the enzootic cycle by calculating basic reproduction number (*R*_0_) of TBE infection.

**Fig 3:**
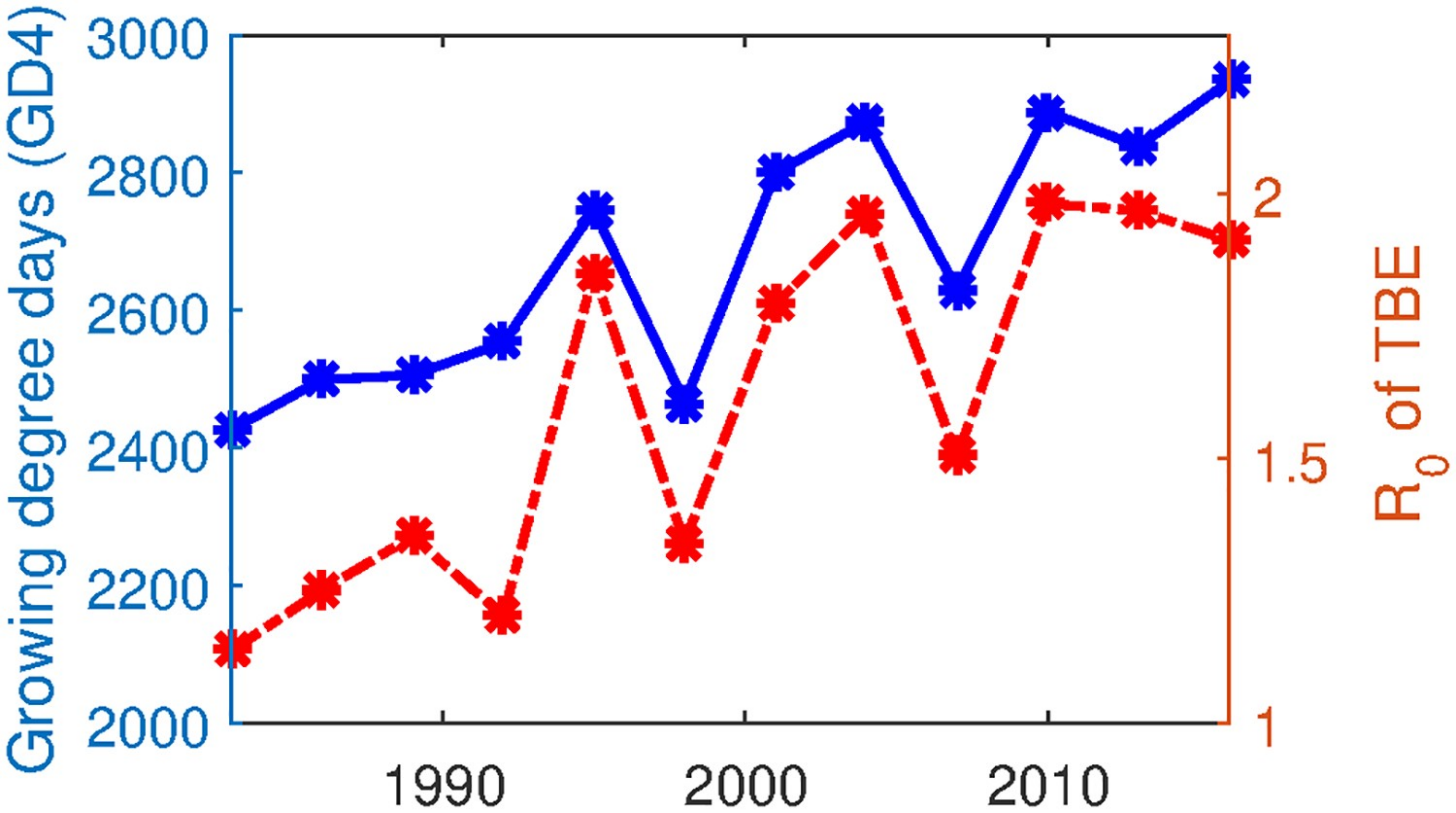
Growing degree days and estimated basic reproduction number of TBE in Szombathely from 1980 to 2015. Solid blue line depicts 3-year average of GD4, sum of the daily mean temperatures exceeding 4°C. Dashed red line shows the basic reproduction numbers calculated at every 3 years from 1980 to 2016. Parameter values used for simulation are listed in Table 3, together with the estimated parameters at the best fit in Fig 2.

The calculation is detailed in S1 Appendix. Fig 3 shows the estimated *R*_0_ and the growing degree days in Szombathely. Between year of 1980 and 2015, R_0_ of TBE is estimated to be between 1.13-1.98. The estimated values are similar to the ones obtained from a modeling study in [23] and the study in a nearby region Borska nizina in Slovakia [7], where R_0_ ranged between 0.85 to 3.27. Sensitivity of *R*_0_ with respect to the estimated parameters are provided in S1 Appendix.

### Trend

**Fig 4:**
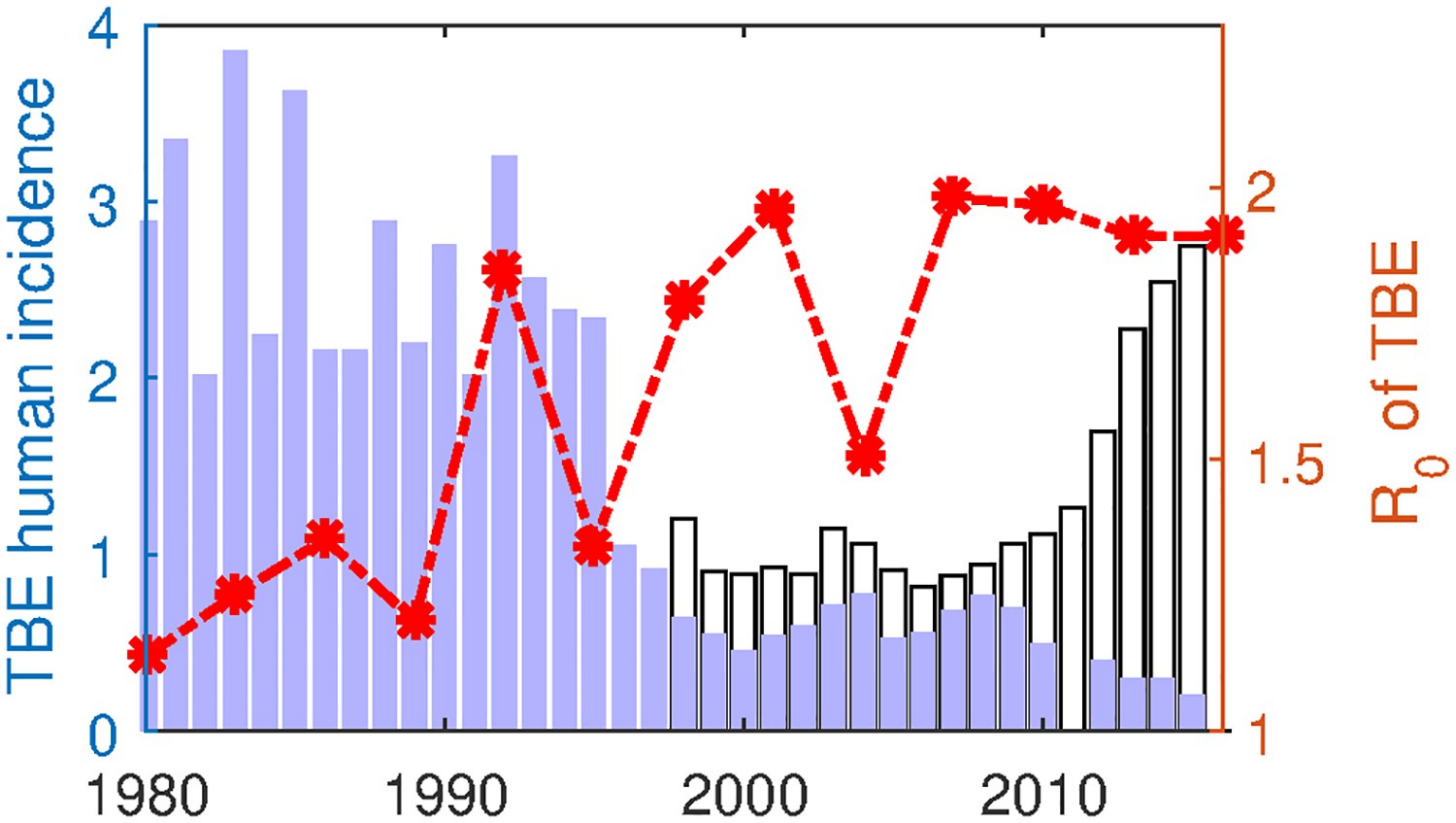
TBE incidence data and estimated basic reproduction number. Blue bars represent TBE incidence (per 100000 people) in Hungary between 1980-2014 [38, 65, 66], with missing data in 2011. White bar shows the expected human case report based on the fitting result. Red line is the estimated *R*_0_. Parameter values used for computing *R*_0_ is in Table 3, together with the estimated parameters at the best fit in Fig 2.

To see the trend of the transmission potential of TBEV in the enzootic cycle with respect to the change of temperatures, we computed *R*_0_ by assuming that (3) is a periodic system with a period of three years taking account of the average tick’s life cycle. We observe the increasing trend in both the growing degree days and the basic reproduction number. Our result clearly shows increase of the transmission potential of TBEV in the enzootic cycle along with the increased temperature between 1980 to 2013. This is in contrast with the observation that TBE human incidence rate has dropped in mid-1990s, see Fig 4. This discrepancy between transmission risk of TBEV in the ecological tick-host cycle and the TBE human case reporting indicates that other public health interventions have been effective in preventing human infection from a large pool of infected tick, unless the reporting rate of TBE has dropped since mid-1990s.

### Further quantification of co-feeding transmission

**Fig 5:**
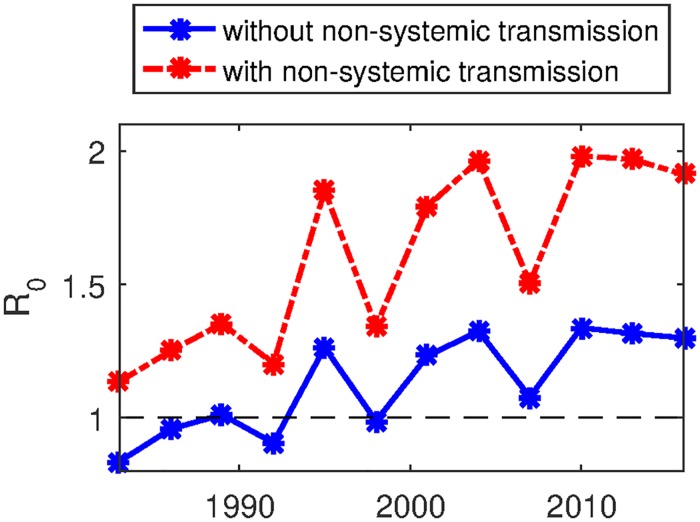
Basic reproduction number of TBE with and without non-systemic transmission. Blue and red lines show the basic reproduction numbers, including and excluding non-systemic transmission, respectively. Parameter values used for simulation are listed in Table 3, together with the estimated parameters at the best fit in Fig 2.

In order to study the significance of the non-systemic transmission route in the transmission of TBEV, we compared the estimated *R*_0_ of TBEV transmission with *R*_0_ of the system which excludes the non-systemic transmission, see Fig 5. As a result, values of *R*_0_ for the system excluding non-systemic transmission lied between 0.84-1.34 in year 1980 to 2015, which are 25%-33% less than the values of *R*_0_ for the system including both systemic and non-systemic transmission route of TBEV (1.13-1.98). The significance of co-feeding transmission estimated in our study is less than the estimates of [25], by which the non-systemic co-feeding pathway is estimated to be 60% greater degree of amplification of TBEV compared with the systemic pathway. Nonetheless, our analysis confirms that co-feeding transmission route is very significant, since the value of *R*_0_ for the system excluding the non-systemic transmission is near the threshold value 1 that determines the sustainability of the disease.

## Discussion

TBE has been a major public health concern for much of the European countries. The risk of human infection depends on the prevalence of the virus spread in the tick-host ecological cycle, and on the tick-human contact that is effective for the disease transmission. Evaluating the human infection needs accurate estimation of the tick-host transmission, the tick population dynamics highly regulated by the environmental and climatic conditions, and the human case reporting given infection probability which is quite stochastic, all involving substantial uncertainties in ecological, epidemiological and environmental parameters. Here we developed a deterministic TBEV transmission model, coupled with a stochastic human case reporting given infection formulation, so that the relatively limited TBE incidence data and relatively complete climate data in the considered region (Hungary) can be used in conjunction with this cascade coupled model to infer key parameters aforementioned.

TBE incidence in Hungary has shown dramatic decrease in 1997. The reason may be a result of under-reporting followed by decreased serological examination or a result of public vaccination in early 1990s [10, 11]. Whether or not the decrease in TBE incidence reflects the actual decrease in human TBE infection level, it seems probable that TBEV transmission level has not been decreased in ecological cycle. The studies on the seropositivity of animals in 1960 to 1970 and 2005 support the argument [12, 13] that the prevalence of the TBEV has not decreased in the hosts. Our results is in line with this argument. The model simulations do not show significant decrease in the basic reproduction number of TBEV or the expected TBE cases between 1980 to 2015. One factor which has contributed to the decline of the incidence is the use of vaccines which protects human from infection upon tick-human contact. The annual TBE cases in a neighboring country Austria has also decreased, while the incidence in the unvaccinated population remains to be unchanged compared with the incidence in the pre-vaccination era [67]. In comparison, there is no vaccine available against Lyme disease transmitted by *Ixodes* ticks and there has been an increasing trend of Lyme disease case in Hungary since 1998 [68]. Our simulations suggest instead that the basic reproduction number in the tick-host transmission cycle has been increasing along with the increasing temperature. In addition, in 2007, the massive use of pesticides to control ticks has stopped due to regulations. This may have further increased the transmission potential of TBEV in the enzootic cycle by allowing better tick survival [69, 70].

We have also observed the significance of non-systemic transmission route on the TBEV transmission. This observation is consistent with the result from the early modeling study [23]. To our knowledge, this is the first modeling study which incorporates explicitly both seasonal dependence and the non-systemic transmission pathway in a single compartmental model setting. By considering seasonal dependence together with the non-systemic transmission pathway, we can examine the seasonal factors in the non-systemic transmission of the virus. For example, non-systemic transmission is only possible when both infective ticks and susceptible ticks are actively questing and the questing activity of ticks shows strong seasonal dependence. It should be mentioned that in our formulation of non-systemic transmission, we assumed that feeding ticks are equally distributed in all hosts. It has been observed that few hosts are attached with the most of the co-feeding ticks [25]. Also, the spatiotemporal distance between co-feeding ticks, which affects the transmissibility, is also neglected in our model formulation. Modification of the model is required to study the effect on disease dynamics of tick distribution over hosts, and the effect of spatiotemporal proximity of feeding ticks on hosts.

We quantified tick-to-host and tick-to-human infections in Hungary. The developed tick-borne disease transmission model and the methodologies we designed is expected to be adopted in the future studies assessing immunization programs for TBE in Hungary.

## Supporting information

S1 Appendix Interpretation of parameter *p*, normalized TBE transmission model, *R*_0_ computation, statistical inference. (PDF)
