# Supplementary material for: Assessing systemic and non-systemic transmission risk of tick-borne encephalitis virus in Hungary
Source: PLoS One. 2019 Jun 4;14(6):e0217206. doi: 10.1371/journal.pone.0217206 (PMC6548428; doi:10.1371/journal.pone.0217206)
Supplement: S1 Appendix — (PDF) [file pone.0217206.s001.pdf]

Supporting Information S1 Appendix

Title: Assessing Systemic and Non-systemic Transmission Risk of Tick-borne Encephalitis Virus in Hungary

Authors: Kyeongah Nah, Felicia Maria G. Magpantay, Ákos Bede-Fazekas, Gergely Röst, Attila János Trájer, Xiaotian Wu, Xue Zhang, Jianhong Wu

1 Interpretation of parameter p

Let $A_{egg}(t)$ be the number of egg-laying adults. Assuming that all egg-laying adults die immediately after oviposition period but not during the period,

$$A_{egg}(t) = \int_{t-\nu}^{\nu} \eta d_{pop}(u) A_e(u) du \approx \nu \eta d_{pop}(t) A_e(t),$$

where ν is the average duration of the oviposition period. Then,

$$\begin{aligned} E'(t) &= p \cdot \eta d_{pop}(t) A_e(t) \cdot e^{-\omega \cdot \eta d_{pop}(t) A_e(t)} - d_{el}(t)E(t) - \mu_e E(t) \\ &= \frac{p}{\nu} A_{egg}(t) e^{-\omega A_{egg}(t)} - d_{el}(t)E(t) - \mu_e E(t), \end{aligned}$$

where $\frac{p}{\nu}$ is the daily birth rate of an egg-laying adult when the population is nearly zero and p is the number of eggs laid by an egg-laying adult during the oviposition period when the population is nearly zero.

2 Normalized TBE transmission model

We normalize the system

$$
\begin{cases}
L_q'(t) = d_{el}(t)E(t) - \alpha_l(t)L_q(t) - \mu_{ql}L_q(t), \\
L_{es}'(t) = (1-\delta(N_{qi}(t),H))\left((1-\beta_{hl})\dfrac{H_i(t)}{H} + \dfrac{H-H_i(t)}{H}\right) f_l\alpha_l(t)L_q(t) - d_{ln}(t)L_{es}(t) - \mu_{el}L_{es}(t), \\
L_{ei}'(t) = \delta(N_{qi}(t),H)\left((1-\beta_{hl})\dfrac{H_i(t)}{H} + \dfrac{H-H_i(t)}{H}\right) f_l\alpha_l(t)L_q(t) \\
\qquad + \beta_{hl}f_l\alpha_l(t)L_q(t)\dfrac{H_i(t)}{H} - d_{ln}(t)L_{ei}(t) - \mu_{el}L_{ei}(t), \\
N_{qs}'(t) = d_{ln}(t)L_{es}(t) - \alpha_n(t)N_{qs}(t) - \mu_{qn}N_{qs}(t), \\
N_{qi}'(t) = d_{ln}(t)L_{ei}(t) - \alpha_n(t)N_{qi}(t) - \mu_{qn}N_{qi}(t), \\
N_{es}'(t) = (1-\delta(N_{qi}(t),H))\left((1-\beta_{hn})\dfrac{H_i(t)}{H} + \dfrac{H-H_i(t)}{H}\right) f_n\alpha_n(t)N_{qs}(t) - d_{na}(t)N_{es}(t) - \mu_{en}N_{es}(t), \\
N_{ei}'(t) = \delta(N_{qi}(t),H)\left((1-\beta_{hn})\dfrac{H_i(t)}{H} + \dfrac{H-H_i(t)}{H}\right) f_n\alpha_n(t)N_{qs}(t) \\
\qquad + \beta_{hn}f_n\alpha_n(t)N_{qs}(t)\dfrac{H_i(t)}{H} + f_n\alpha_n(t)N_{qi}(t) - d_{na}(t)N_{ei}(t) - \mu_{en}N_{ei}(t), \\
A_{qs}'(t) = d_{na}(t)N_{es}(t) - \alpha_a(t)A_{qs}(t) - \mu_{qa}A_{qs}(t), \\
A_{qi}'(t) = d_{na}(t)N_{ei}(t) - \alpha_a(t)A_{qi}(t) - \mu_{qa}A_{qi}(t), \\
A_e'(t) = f_a\alpha_a(t)(A_{qs}(t) + A_{qi}(t)) - d_{pop}(t)A_e(t) - \mu_{ea}A_e(t), \\
E'(t) = p\cdot\eta d_{pop}(t)A_e(t)\cdot e^{-\omega\cdot\eta d_{pop}(t)A_e(t)} - d_{el}(t)E(t) - \mu_e E(t), \\
H_s'(t) = bH - \beta_{nh}\alpha_n(t)\dfrac{N_{qi}(t)}{H}H_s(t) - bH_s(t), \\
H_i'(t) = \beta_{nh}\alpha_n(t)\dfrac{N_{qi}(t)}{H}H_s(t) - \gamma H_i(t) - bH_i(t).
\end{cases}
$$

by introducing variables $\widetilde{L_q}(t) = \frac{L_q(t)}{H}$, $\widetilde{L_{es}}(t) = \frac{L_{es}(t)}{H}$, $\widetilde{L_{ei}}(t) = \frac{L_{ei}(t)}{H}$, $\widetilde{N_{qs}}(t) = \frac{N_{qs}(t)}{H}$, $\widetilde{N_{qi}}(t)(t) = \frac{N_{qi}(t)}{H}$, $\widetilde{N_{es}}(t) = \frac{N_{es}(t)}{H}$, $\widetilde{N_{ei}}(t) = \frac{N_{ei}(t)}{H}$, $\widetilde{A_{qs}}(t) = \frac{A_{qs}(t)}{H}$, $\widetilde{A_{qi}}(t) = \frac{A_{qi}(t)}{H}$, $\widetilde{E}(t) = \frac{E(t)}{H}$, $\widetilde{H_s}(t) = \frac{H_s(t)}{H}$

and $\widetilde{H_i}(t) = \frac{H_i(t)}{H}$. The resulting system is

$$
\begin{cases}
\widetilde{L_q}'(t) = d_{el}(t)\widetilde{E}(t) - \alpha_l(t)\widetilde{L_q}(t) - \mu_{ql}\widetilde{L_q}(t), \\
\widetilde{L_{es}}'(t) = (1-\delta(\widetilde{N_{qi}}))((1-\beta_{hl})\widetilde{H_i}(t) + (1-\widetilde{H_i}(t)))f_l\alpha_l(t)\widetilde{L_q}(t) - d_{ln}(t)\widetilde{L_{es}}(t) - \mu_{el}\widetilde{L_{es}}(t), \\
\widetilde{L_{ei}}'(t) = \delta(\widetilde{N_{qi}})((1-\beta_{hl})\widetilde{H_i}(t) + (1-\widetilde{H_i}(t)))f_l\alpha_l(t)\widetilde{L_q}(t) + \beta_{hl}f_l\alpha_l(t)\widetilde{L_q}(t)\widetilde{H_i}(t) - d_{ln}(t)\widetilde{L_{ei}}(t) - \mu_{el}\widetilde{L_{ei}}(t), \\
\widetilde{N_{qs}}'(t) = d_{ln}(t)\widetilde{L_{es}}(t) - \alpha_n(t)\widetilde{N_{qs}}(t) - \mu_{qn}\widetilde{N_{qs}}(t), \\
\widetilde{N_{qi}}'(t) = d_{ln}(t)\widetilde{L_{ei}}(t) - \alpha_n(t)\widetilde{N_{qi}}(t) - \mu_{qn}\widetilde{N_{qi}}(t), \\
\widetilde{N_{es}}'(t) = (1-\delta(\widetilde{N_{qi}}))\left((1-\beta_{hn})\widetilde{H_i}(t) + (1-\widetilde{H_i}(t))\right) f_n\alpha_n(t)\widetilde{N_{qs}}(t) - d_{na}(t)\widetilde{N_{es}}(t) - \mu_{en}\widetilde{N_{es}}(t), \\
\widetilde{N_{ei}}'(t) = \delta(\widetilde{N_{qi}})\left((1-\beta_{hn})\widetilde{H_i}(t) + (1-\widetilde{H_i}(t))\right) f_n\alpha_n(t)\widetilde{N_{qs}}(t) \\
\qquad + \beta_{hn}f_n\alpha_n(t)\widetilde{N_{qs}}(t)\widetilde{H_i}(t) + f_n\alpha_n(t)\widetilde{N_{qi}}(t) - d_{na}(t)\widetilde{N_{ei}}(t) - \mu_{en}\widetilde{N_{ei}}(t), \\
\widetilde{A_{qs}}'(t) = d_{na}(t)\widetilde{N_{es}}(t) - \alpha_a(t)\widetilde{A_{qs}}(t) - \mu_{qa}\widetilde{A_{qs}}(t), \\
\widetilde{A_{qi}}'(t) = d_{na}(t)\widetilde{N_{ei}}(t) - \alpha_a(t)\widetilde{A_{qi}}(t) - \mu_{qa}\widetilde{A_{qi}}(t), \\
\widetilde{A_e}'(t) = f_a\alpha_a(t)(\widetilde{A_{qs}}(t) + \widetilde{A_{qi}}(t)) - d_{pop}(t)\widetilde{A_e}(t) - \mu_{ea}\widetilde{A_e}(t), \\
\widetilde{E}'(t) = p\cdot\eta d_{pop}(t)\widetilde{A_e}(t)\cdot e^{-\widetilde{\omega}\cdot\eta d_{pop}(t)\widetilde{A_e}(t)} - d_{el}(t)\widetilde{E}(t) - \mu_e\widetilde{E}(t), \\
\widetilde{H_s}'(t) = b - \beta_{nh}\alpha_n(t)\widetilde{N_{qi}}(t)\widetilde{H_s}(t) - b\widetilde{H_s}(t), \\
\widetilde{H_i}'(t) = \beta_{nh}\alpha_n(t)\widetilde{N_{qi}}(t)\widetilde{H_s}(t) - \gamma\widetilde{H_i}(t) - b\widetilde{H_i}(t),
\end{cases}
$$

where $\widetilde{\omega} = \omega H$ and $\delta(\widetilde{N_{qi}}) = 1 - (1-c)^{T_f\alpha(t)\widetilde{N_{qi}}}$.

3 R_0 of TBE transmission model

We linearize the infected subsystem of (2) at the disease-free periodic solution

$(\hat{L_q}(t), \hat{L_{es}}(t), \hat{0}, \hat{N_{qs}}(t), \hat{0}, \hat{N_{es}}(t), \hat{0}, \hat{A_{qs}}(t), \hat{0}, \hat{A_e}(t), \hat{H_s}(t), \hat{0})$ and obtain

$$
\begin{bmatrix} L'_{ei}(t) & N'_{qi}(t) & N'_{ei}(t) & A'_{qi}(t) & H'_i(t) \end{bmatrix}^T = (F(t) - V(t)) \begin{bmatrix} L_{ei}(t) & N_{qi}(t) & N_{ei}(t) & A_{qi}(t) & H_i(t) \end{bmatrix}^T,
$$

where $F(t)$ and $V(t)$ are time periodic matrix valued functions representing reproduction of new

infections and transition between compartments taking forms as

$$F(t) = \begin{pmatrix} 0 & -T_f\alpha_n(t)\ln(1-c)f_l\alpha_l(t)\hat{L_q}(t) & 0 & 0 & \beta_{hl}f_l\alpha_l(t)\hat{L_q}(t) \\ 0 & 0 & 0 & 0 & 0 \\ 0 & -T_f\alpha_n(t)\ln(1-c)f_n\alpha_n(t)\hat{N_{qs}}(t) & 0 & 0 & \beta_{hn}f_n\alpha_n(t)\hat{N_{qs}}(t) \\ 0 & 0 & 0 & 0 & 0 \\ 0 & \beta_{nh}\alpha_n(t) & 0 & 0 & 0 \end{pmatrix},$$

$$V(t) = \begin{pmatrix} d_{ln}(t)+\mu_{el} & 0 & 0 & 0 & 0 \\ -d_{ln}(t) & \alpha_n(t)+\mu_{qn} & 0 & 0 & 0 \\ 0 & -f_n\alpha_n(t) & d_{na}(t)+\mu_{en} & 0 & 0 \\ 0 & 0 & -d_{na}(t) & \alpha_a(t)+\mu_{qa} & 0 \\ 0 & 0 & 0 & 0 & \gamma+b \end{pmatrix}.$$

The basic reproduction number is the spectral radius of the next generation operator defined with the above $F(t)$ and $V(t)$.

4 Statistical Inference

Initial Conditions

We use Latin Hypercube Sampling to choose parameters and compare the likelihoods at each parameter sets. During the Latin Hypercube Sampling process, initial values of the variables are also sampled. With each of the sampled parameter sets, we obtained the numerical solution with the initial time corresponding to the year of 1901 allowing long enough time for the states to approach a periodic solution prior to the time corresponding to the earliest year of the data points (1998).

Likelihood Profiles

By the maximum likelihood estimation, we estimate the unknown parameters: probabilities of successful feeding (f_l, f_n, f_a), host-attaching rate of actively questing ticks (λ_l, λ_n, λ_a), degree of density dependent fecundity (ω), host recovery rate (γ), mortality of hosts (b), relative ratio between nymphs and adults for the human attachments (κ), the degree of temperature dependency on the human-attaching rate of nymphs (α), the minimum temperature for the activity of questing larvae (m_l) and the reporting probability (ρ).

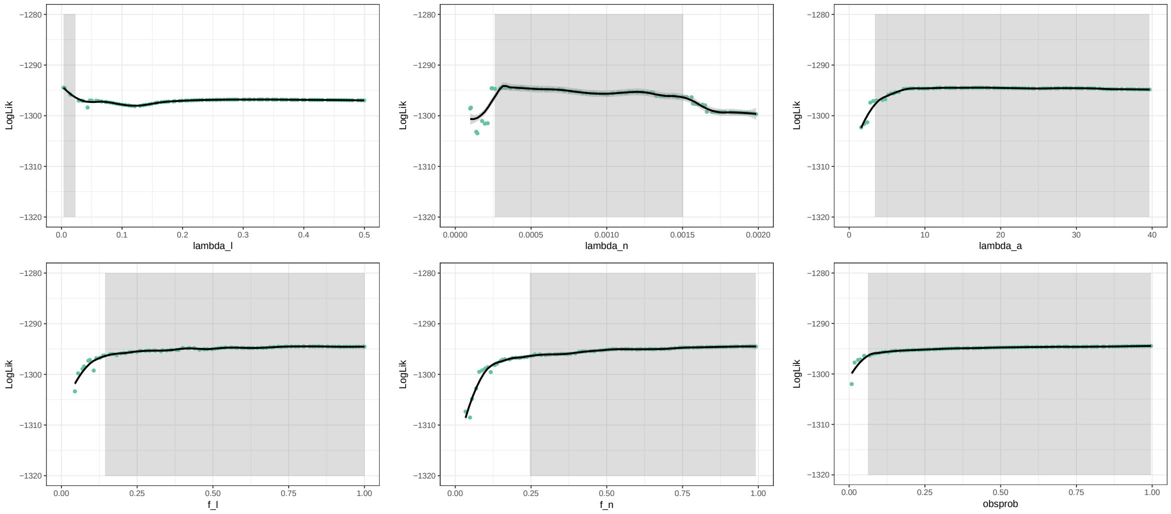

Figure 4.1: **Plots of the log-likelihood profiles corresponding to the maximal likelihood estimates.** The shaded parts correspond to the 95% confidence intervals of the parameter values which yield the highest likelihood values. From the top-left panel, we present the likelihood profiles corresponding to parameters λ_l, λ_n, λ_a, f_l, f_n and ρ.

We check the convergence by computing the likelihood profiles. During this process we have observed a strong correlation between parameter α (a degree of temperature dependency on the human-attaching rate of nymphs) and ρ (observation probability). To resolve non-identifiability, we have fixed the value of α as a constant value and estimated other unknown parameters. The value (α=1660) was chosen by the maximum likelihood method. Figure 4.1 shows the likelihood profiles of the maximal likelihood estimates.

Sensitivity Analysis

In order to parameterize TBE virus transmission model and estimate R_0, we rank the likelihood and choose the parameter set which gives the maximum likelihood. As we observe from Fig. 4.1, there are more than one parameter set that gives maximized likelihood. To ensure the result (R_0) is not sensitive to the estimations, we checked the sensitivity of R_0 with respect to the estimated parameters. The sensitivity analysis is summarized in Fig. 4.2. Note that what matters for our calculation is $\alpha_l(t) = p_l(t) \times \lambda_l$ and hence there is a clear positive relation between the estimated λ_l (highest point of the step function $\alpha_l(t)$) and m_l as shown in Figure 4.3.

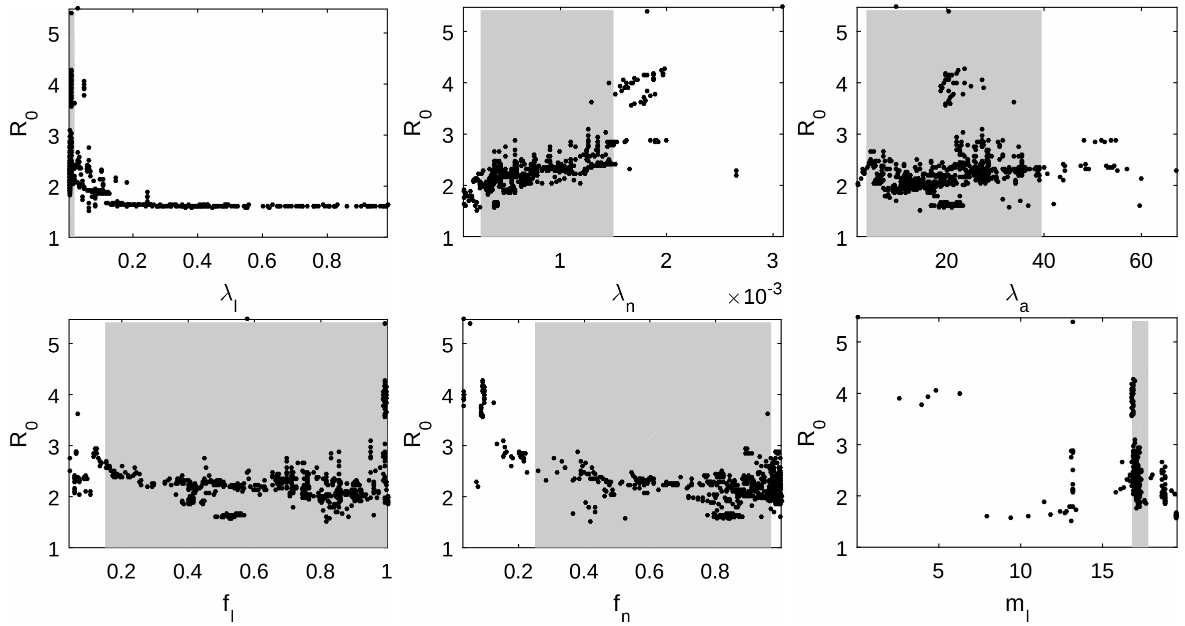

Figure 4.2: **Sensitivity of R_0 with respect to the maximal likelihood estimates.** Panels in upper and lower sides are the scatter plots of the parameters and R_0. Among top 8355 set parameters which yield highest likelihoods, 1000 set of sample parameters are drawn by a probability sampling method by weighting the parameter sets according to their normalized likelihoods. The shaded parts correspond to the 95% confidence intervals of the parameter values which yield the highest likelihood values. To compute R_0 of the 3-year periodic system, we used the temperature data in the year of 2010-2013.

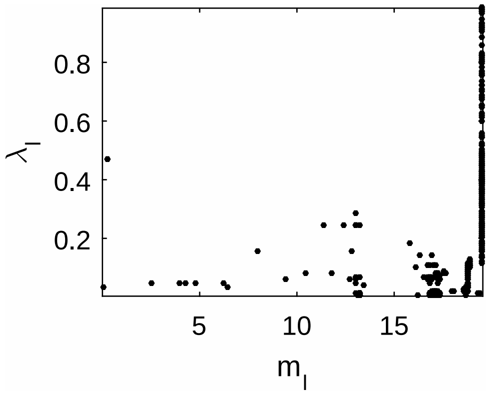

Figure 4.3: **Relation between m_l and λ_l.** The scatter plot of the estimated parameters m_l and λ_l shows the positive relation between the two estimates.
